# Association between Serum Vitamin D Metabolites and Metabolic Function in Healthy Asian Adults

**DOI:** 10.3390/nu12123706

**Published:** 2020-11-30

**Authors:** Cherlyn Ding, Zhiling Chan, Yu Chung Chooi, John Choo, Suresh Anand Sadananthan, Navin Michael, Sambasivam Sendhil Velan, Melvin Khee-Shing Leow, Faidon Magkos

**Affiliations:** 1 Singapore Institute for Clinical Sciences (SICS), Department of Human Development, Agency for Science, Technology and Research (A*STAR), Singapore 117609, Singapore; dingcherlyn@gmail.com (C.D.); zhilingchan@hotmail.com (Z.C.); cy338@hotmail.com (Y.C.C.); jc29128@gmail.com (J.C.); suresh@sics.a-star.edu.sg (S.A.S.); navin_michael@sics.a-star.edu.sg (N.M.); sendhil_velan@sbic.a-star.edu.sg (S.S.V.); melvin_leow@sics.a-star.edu.sg (M.K.-S.L.); 2 Singapore Bioimaging Consortium, Agency for Science, Technology and Research (A*STAR), Singapore 138667, Singapore; 3 Department of Physiology, Yong Loo Lin School of Medicine, Singapore 117593, Singapore; 4 Department of Medicine, Yong Loo Lin School of Medicine, National University of Singapore, Singapore 119077, Singapore; 5 Metabolic Disorders Research Program, Lee Kong Chian School of Medicine, Nanyang Technological University, Singapore 636921, Singapore; 6 Cardiovascular and Metabolic Disorders Program, Duke-NUS Medical School, Singapore 169857, Singapore; 7 Department of Endocrinology, Tan Tock Seng Hospital, Singapore 308433, Singapore; 8 Department of Nutrition, Exercise and Sports, University of Copenhagen, Frederiksberg C, 1958 København, Denmark

**Keywords:** vitamin D metabolites, metabolic dysfunction, glucose homeostasis

## Abstract

The association between low vitamin D status and the development of type 2 diabetes mellitus is well established; however, intervention trials that increased serum vitamin D (through ultraviolet B exposure or dietary supplementation) provide mixed outcomes. Recent evidence suggests that metabolites directly related to vitamin D receptor activation—1α,25-dihydroxyvitamin D_3_ and 24*R*,25-dihydroxyvitamin D_3_—may be better markers of vitamin D repletion status. We tested the hypothesis that a vitamin D metabolite (VDM) index, calculated as the sum of normalized fasting serum concentrations of 1α,25-dihydroxyvitamin D_3_ and 24*R*,25-dihydroxyvitamin D_3_, is associated with metabolic function. We measured subcutaneous and visceral adipose tissue volume, intrahepatic triglyceride content, maximum oxygen uptake, insulin sensitivity (4 h hyperinsulinemic-euglycemic clamp), and insulin secretion (3 h meal tolerance test with mathematical modeling) and calculated the VDM index in 65 healthy Asian adults. Subjects with a low VDM index had lower peripheral insulin sensitivity and beta-cell function compared to subjects with a high VDM index (both *p* < 0.05), matched for age, sex, BMI, and serum 25-hydroxyvitamin D_3_. Serum 25-hydroxyvitamin D_3_ was not associated with peripheral insulin sensitivity or beta-cell function. Our results suggest that, rather than enhancing vitamin D substrate availability, upregulation of vitamin D action is more likely to lead to improvements in glucose homeostasis.

## 1. Introduction

Hypovitaminosis D, or vitamin D deficiency, is traditionally associated with the development of rickets and musculoskeletal disorders. It is estimated that one billion people worldwide are at risk of vitamin D deficiency, with 30–60% of both children and adults in the United States, Europe, South America, the Middle East, and the Far East reported to be vitamin D deficient or insufficient. The major causes of vitamin D deficiency are the lack of exposure to sunlight and reduced intake from dietary sources [1].

In recent times, significant links between vitamin D deficiency and metabolic diseases, such as diabetes, obesity, and metabolic syndrome, have also been established [2,3]. Based on the strong epidemiologic relationship between low vitamin D_3_ levels and metabolic dysfunction, clinical trials that intervened to increase plasma vitamin D_3_ levels through ultraviolet B (UVB) exposure and dietary supplementation have been undertaken to evaluate potential therapeutic effects. Remarkably, only a small proportion of these studies have reported significant positive outcomes [4,5].

Recent advancements in mass spectrometry-based clinical detection of plasma metabolites have allowed for the accurate detection of 1α,25-dihydroxyvitamin D_3_ (1,25(OH)_2_D_3_), the active metabolite of vitamin D and specific ligand for the vitamin D receptor (VDR), and 24*R*,25-dihydroxyvitamin D_3_ (24,25(OH)_2_D_3_), the major product of 25-hydroxyvitamin D_3_ (25(OH)D_3_) catabolism, in human blood. Here, we postulate that the measurement of these metabolites could better indicate vitamin D repletion compared to 25(OH)D_3_, which is more widely measured [6,7]. This is because of the direct relationships of these metabolites to VDR activation: the expression of CYP24A1 (24-hydroxylase), which converts 25(OH)D_3_ to 24,25(OH)_2_D_3_, is regulated in part by activation of VDR, and VDR activation is dependent upon 1,25(OH)_2_D_3_ binding [8]. We hypothesized that vitamin D metabolism is altered in metabolically unhealthy subjects, which could help explain the low rates of efficacy in vitamin D supplementation trials.

Accordingly, we tested the hypothesis that adults with low summed levels of serum 1,25(OH)_2_D_3_ and 24,25(OH)_2_D_3_, which we termed as the “vitamin D metabolite” (VDM) index, have inferior metabolic function compared to age-, sex-, BMI-, and vitamin D_3_-matched controls with a high VDM index.

## 2. Materials and Methods

### 2.1. Subjects

Seventy-six lean and apparently healthy Asian subjects (age 21–65 years old; 51% female, BMI < 25 kg/m^2^) were screened for this study. Subjects had no prior history of metabolic disease, and all had nondiabetic fasting blood glucose levels (upper limit: 102 mg/dL) and HbA1c (upper limit: 6.2% or 44 mmol/mol) at screening. Subjects were excluded from the study if they were using medications known to affect metabolic function, used tobacco products and consumed alcohol regularly, had significant organ system dysfunction or disease, or experienced recent weight loss or gain (≥5% over the past 6 months). Ethics approval was obtained from the Domain Specific Review Board of the National Healthcare Group in Singapore (NHG DSRB Ref: 2016/00222), and informed consent was obtained from subjects prior to enrolment. Ten subjects dropped out before completing all tests, and one subject was excluded from the analysis due to a technical problem with the intrahepatic triglyceride (IHTG) measurement.

### 2.2. Body Composition and Maximal Oxygen Uptake

The methods for measuring fat mass and fat-free mass (dual-energy X-ray absorptiometry), visceral adipose tissue (VAT) and subcutaneous abdominal adipose tissue (SAT) (magnetic resonance imaging), IHTG content (magnetic resonance spectroscopy), and maximum oxygen uptake (VO_2max_) (graded exercise test on a cycloergometer) have been described previously in detail [9,10,11].

### 2.3. Metabolic Testing

Metabolic tests were carried out during two separate visits to the Clinical Nutrition Research Centre (CNRC). Before the visits, subjects were instructed to abstain from caffeine and alcohol consumption the day before and to avoid eating atypically or performing any strenuous exercise on the preceding 3 days. Subjects arrived at the CNRC in the morning after an overnight fast. Whole-body insulin sensitivity was determined by using a 4 h hyperinsulinemic-euglycemic clamp procedure, and beta-cell responsivity and postprandial insulin secretion rates were evaluated by using oral minimal modelling after ingestion of a standardized mixed meal, as previously described [11,12].

### 2.4. Clinical Chemistry

A bedside glucose analyzer (YSI 2300 Stat Plus; YSI Life Sciences, Yellow Spring, OH, USA) was used to measure plasma glucose concentration. Plasma insulin and C-peptide concentrations were measured by using commercially available electrochemiluminescence assays (Cobas e411 immunochemistry analyzer; Roche Diagnostics, Indianapolis, IN, USA). Serum-free fatty acid (FFA) concentration was determined by using an enzymatic colorimetric method (Wako Diagnostics, Mountain View, CA, USA). Total plasma triglyceride, total cholesterol, low-density lipoprotein (LDL), and high-density lipoprotein (HDL) cholesterol concentrations were determined by standard methods at the National University Hospital (NUH) Referral Laboratory (accredited by the College of American Pathologists). 25(OH)D_3_, 24,25(OH)_2_D_3_, and 1,25(OH)_2_D_3_ concentrations were determined by ultra-high-performance liquid chromatography-tandem mass spectrometry (UHPLC-MS/MS). Acetonitrile, formic acid, methanol, methylamine, and 4-(4-(2-Azidoethoxy)phenyl)-1,2,4- triazolidine-3,5-dione (PTAD) were sourced from Merck KGaA (Darmstadt, Germany). Standards and deuterated internal standards for 25(OH)D_3_, 24,25(OH)_2_D_3_, and 1,25(OH)_2_D_3_ were sourced from Isosciences (Ambler, PA, USA) and prepared in acetonitrile. Protein precipitation and solid-phase extraction were carried out according to the method of Ding et al. [13]. Briefly, serum samples were inoculated with 10 μL of internal standard solution, followed by solid phase extraction (Strata-X extraction columns, Phenomenex, Torrance, CA, USA), elution with acetonitrile, and vacuum-drying. Thereafter, sample derivatization was carried out by pipetting 200 μL of 2 mg/mL PTAD in acetonitrile into the dried samples and incubated before injection into LC-MS. Analysis of vitamin D metabolites was performed on an ACQUITY UPLC/Xevo TQ-S (Waters Corporation, Milford, MA, USA) equipped with an electrospray source operating in positive (ESI+) mode. The source temperature was set at 150 °C with a cone gas flow of 150 L/h, a desolvation gas flow of 900 L/h, and a desolvation gas temperature of 500 °C. The capillary voltage was set to 3.50 kV and the cone voltage at 80 V. Samples (10 μL) were injected into a 50 mm × 2.1 mm, 1.7 μm BEH C18 column (Waters) maintained at 40 °C. Elution was performed at a linear gradient of 35–2.0% A over 0.5–3.5 min, held for 1 min, 2.0–35% A over 0.1 min, and re-equilibrated at 35% A over 1.4 min (Mobile Phase A: water + 0.1% formic acid + 5 mM methylamine, Mobile Phase B: methanol + 0.1% formic acid). The column flow rate was set at 0.3 mL/min.

### 2.5. Calculations

Insulin sensitivity: Insulin-mediated whole-body glucose disposal (M-value, in mg of glucose per kg fat-free mass per minute) was calculated as the average rate of dextrose infusion during the final 30 min of the clamp when the steady-state was reached. Insulin sensitivity was calculated as the M/I ratio, that is glucose disposal (M-value) divided by the steady-state plasma insulin concentration.

Insulin secretion and beta-cell function: The insulin secretion rate (ISR, in mU/L·min) before and after ingestion of the mixed meal was assessed by using oral minimal model analysis of plasma C-peptide and glucose concentrations (Simulation, Analysis and Modeling Software, SAAM II version 2.3, The Epsilon Group, Charlottesville, VA, USA); this provided static ISR (insulin secretion response to a given glucose concentration), dynamic ISR (insulin secretion response to a given change in glucose concentration), and total ISR (total insulin secretion) [14]. Cumulative postprandial ISRs (in mU/L) were computed as the corresponding areas under the curve (AUC) for 3 h after ingestion of the mixed meal. The disposition index was calculated as the product of insulin sensitivity (M/I ratio) and the static, dynamic, or total insulin secretion rate AUC (mU/L; by mixed meal tolerance test) [15].

VDM index: The VDM index (unitless) was calculated as the sum of normalized (log- transformed) fasting serum concentrations of 1,25(OH)_2_D_3_ and 24,25(OH)_2_D_3_.

### 2.6. Statistical Analysis

All analyses were carried out with SPSS Version 23 (IBM SPSS, Chicago, IL, USA). The Shapiro–Wilk test was used to evaluate the distribution of the data. Results are reported as the mean ± SD for normally distributed data and compared between groups with the Student unpaired *t*-test or median and quartiles for non-parametric data and compared between groups with the Mann–Whitney U-test. Correlation analysis was used to test for associations between variables of interest (Pearson’s correlation coefficient for parametric distributions and Spearman’s correlation coefficient for non-parametric distributions). To compare the metabolic characteristics of subjects with a low versus high VDM index, subjects (beginning from the subject with the lowest VDM index) were matched for age, sex, BMI, and vitamin D exposure (reflected by the serum 25(OH)D_3_ concentration) to subjects with at least a 10% or higher VDM index. Adjustment for potential confounders (e.g., age, sex, BMI) was performed as needed. Statistical significance was set at *p* ˂ 0.05.

## 3. Results

### 3.1. Vitamin D Metabolites, Body Composition, and Cardiometabolic Factors

**Figure 1:**
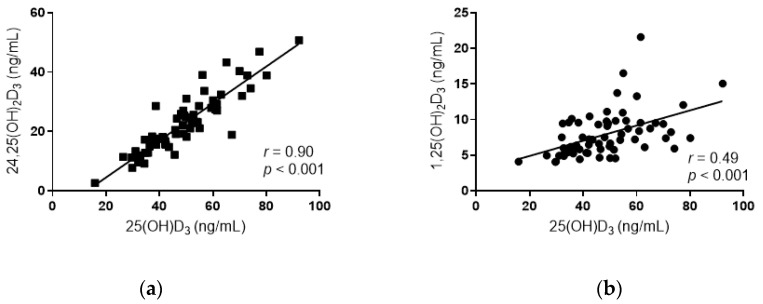
Relationship between the concentrations of *24R*,25-dihydroxyvitamin D_3_ (24,25(OH)_2_D_3_) (**a**) and 1α,25-dihydroxyvitamin D_3_ (1,25(OH)_2_D_3_) (**b**) with 25-hydroxyvitamin D_3_ in all subjects (*n* = 65). Pearson correlation coefficients (*r*) and *p*-values are shown.

Serum 25(OH)D_3_ concentration showed strong positive correlations with both 24,25(OH)_2_D_3_ concentration (*r* = 0.90, *p* < 0.001; Figure 1a) and 1,25(OH)_2_D_3_ concentration (*r* = 0.49, *p* < 0.001; Figure 1b). The VDM index was significantly greater in men than in women (2.26 ± 0.35 and 2.08 ± 0.28, respectively; *p* = 0.024). After adjustment for age, BMI, and sex, the VDM was negatively associated with percent body fat, SAT, waist circumference, and diastolic blood pressure and was positively associated with insulin sensitivity (M/I) and the total disposition index (Table 1). Serum 25(OH)D_3_ concentration was not associated with M/I (*p* = 0.449) or the total disposition index (*p* = 0.14).

### 3.2. Characteristics of Subjects with a Low and a High VDM Index

**Figure 2:**
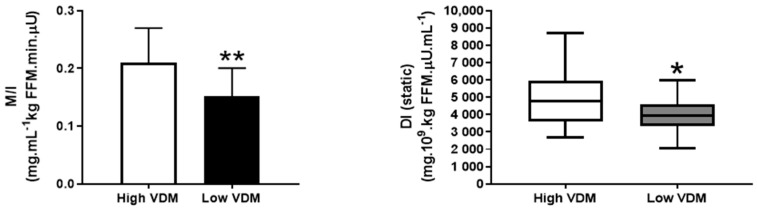

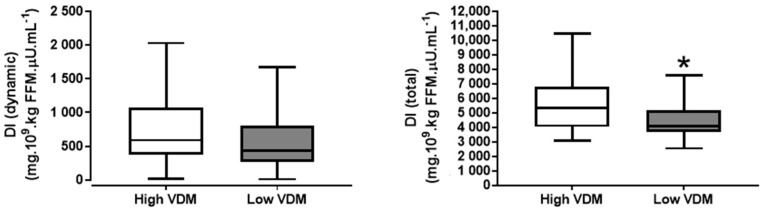
Peripheral insulin sensitivity (**a**) and the static, dynamic, and total (**b**–**d**) disposition index (DI) in the high and low VDM index subjects matched for age, sex, BMI, and vitamin D exposure (serum 25(OH)D_3_). Values are the mean ± SD (**a**) or median and quartiles (**b**–**d**). * *p* < 0.05; ** *p* < 0.01, the value is significantly different from the corresponding value in the high VDM index group.

Out of the 65 subjects analyzed and starting from the subject with the lowest VDM index, we identified 17 subjects (9 males and 8 females) with a low VDM index (mean ± SD: 2.00 ± 0.27) and matched them for age, sex, BMI, and serum 25(OH)D_3_ to 17 subjects with a ≥10% higher VDM index (mean ± SD: 2.32 ± 0.31, *p* = 0.003 vs. low VDM group, Table 2); the difference in the VDM index among matched pairs of subjects ranged from 12% to 27%. The low VDM index group had significantly lower peripheral insulin sensitivity (M/I ratio) and a lower total and static disposition index compared to the high VDM index group (Figure 2). The low VDM index subjects also tended to have greater IHTG content than the high VDM index subjects, although this did not reach statistical significance (Table 2).

## 4. Discussion

Our data suggest that vitamin D status, determined from serum concentrations of 1,25(OH)_2_D_3_ and 24,25(OH)_2_D_3_, is associated with insulin sensitivity and beta-cell function independent of body composition, fat distribution, and vitamin D exposure. This finding adds to current evidence that vitamin D status is important in the maintenance of glucose homeostasis. However, data from our study support the notion that factors other than substrate availability may affect vitamin D action and its role in metabolic function.

Serum 25(OH)D_3_ is the most widely used indicator of vitamin D status; its stability, high relative abundance, and long half-life make it amenable to robust clinical measurement in most clinical and research settings [16]. A committee of the Institute of Medicine determined that individuals may be at risk of vitamin D deficiency at serum 25(OH)D_3_ concentrations <30 nmol/L (<12 ng/mL), while individuals with serum 25(OH)D_3_ concentrations ≥50 nmol/L (≥20 ng/mL) are deemed to have sufficient vitamin D. While these cutoffs represent average concentrations at the population level, they fail to account for the large variation in 25(OH)D_3_ that denotes adequacy at an individual level. To illustrate, many patients have very low 25(OH)D_3_ values without increased parathyroid hormone (PTH) production; conversely, serum concentrations of 25(OH)D_3_ greater than 30 ng/mL are not always associated with PTH suppression [16]. For these reasons, concentrations of 25(OH)D_3_ in serum are generally regarded as a biomarker of vitamin D exposure rather than a biomarker of effect.

1,25(OH)_2_D_3_ and 24,25(OH)_2_D_3_ are closely related compounds present at different stages of vitamin D metabolism. 1,25(OH)_2_D_3_ is formed in the kidney and peripheral tissues by the action of CYP27B1 (1α-hydroxylase) on 25(OH)D_3_ and is the active metabolite of vitamin D_3_ and the specific ligand for the VDR [8]. 24,25(OH)_2_D_3_ is also synthesized from 25(OH)D_3_ by the action of CYP24A1 (24-hydroxylase), an enzyme expressed by vitamin D target tissues in a negative feedback mechanism dependent on VDR binding to 1,25(OH)_2_D_3_ [8]. Its production is generally regarded as the first step in the inactivation of 25(OH)D_3_, thus regulating VDR activation through downstream inhibition of 1,25(OH)_2_D_3_ synthesis from 25(OH)D_3_. It has been proposed that 24,25(OH)_2_D_3_ may be biologically active, but a receptor for this metabolite has not yet been discovered [17].

In our study, 1,25(OH)_2_D_3_ and 24,25(OH)_2_D_3_ correlated strongly with 25(OH)D_3_, similar to observations in previous studies [6,7]. The dose-response relationships between 1,25(OH)_2_D_3,_ 24,25(OH)_2_D_3_, and 25(OH)D_3_ suggest that the serum concentrations or the substrate availability of 25(OH)D_3_ may confound meaningful interpretation of 1,25(OH)_2_D_3_ and 24,25(OH)_2_D_3_ levels as independent indicators of vitamin D signaling activity. Accordingly, previous studies that assessed the ratio of 24,25(OH)_2_D_3_ to 25(OH)D_3_ have shown significant associations with bone mineralization outcomes, suggesting that the proportion of 24,25(OH)_2_D_3_ produced in relation to 25(OH)D_3_ (likely related to the rate of catabolism) reflects vitamin D repletion status [6,7]. Similar to the VDM index, we note significant associations between the ratio of 24,25(OH)_2_D_3_ to 25(OH)D_3_ with metabolic outcomes in our subjects (data not shown), particularly with respect to VAT, IHTG content, and beta-cell function. However, the VDM index showed additional and stronger associations with direct measures of glucose homeostasis independent of vitamin D exposure (as reflected by serum 25(OH)D_3_ concentrations). We speculate the strong associations of the VDM index with insulin sensitivity and beta-cell function were likely because this index indirectly takes into account the rate and extent of conversion of 25(OH)D_3_ to 1,25(OH)_2_D_3_ (1α-hydroxylase activity), as well as the production of 24,25(OH)_2_D_3_ (24-hydroxylase activity; which is upregulated in response to VDR activation). This could render the VDM index more indicative of vitamin D repletion status. In support, 1,25(OH)_2_D_3_ administration was previously linked to improved insulin sensitivity and insulin secretion in human clinical trials [18,19]. The attenuation of inflammatory signaling pathways in hepatocytes and pancreatic beta-cells, as well as the regulation of intracellular Ca^2+^ oscillations in beta-cells (which triggers insulin secretion) have been proposed as possible mechanisms by which beta-cell function improves [20,21,22]. Our findings also suggest that optimal vitamin D metabolism is linked to better insulin sensitivity, which may also contribute to improvements in beta-cell function by reducing the metabolic demand for insulin. Further, the VDM index was associated with static, but not dynamic beta-cell responsivity. This suggests that suboptimal VDR activation may reduce insulin granule translocation and maturation in the beta-cell. In support, VDR activation and hypovitaminosis D have been previously linked to forkhead box protein O1 activity. FoxO1-transgenic mice showed significantly higher *Ins1* and *Ins2* expression in islets, along with a significant induction of *Pdx1*, *MafA*, *FoxA2*, and *Glut2* expression—key transcription factors and genes regulating insulin production, beta-cell glucose sensing, and insulin signaling [23,24].

Serum 25(OH)D_3_ was previously shown to be negatively associated with non-alcoholic fatty liver disease [25,26], and molecular studies suggest it has a mechanistic role in regulating sterol regulatory element-binding protein cleavage-activating protein complex formation in the liver [27]. This relationship was apparent in our dataset, as low VDM index subjects had 2.5-fold higher IHTG content compared to high VDM index subjects (upon comparison of mean values), and IHTG content showed a negative association trend with the VDM index. As liver fat deposition has been suggested to be an independent predictor of beta-cell function [28,29], we further adjusted for IHTG in our analysis and found that the VDM index remained significantly associated with beta-cell function (data not shown). This suggests that lower beta-cell function may be attributed to alterations in vitamin D metabolism, independent of generalized and ectopic adiposity.

Our study has a number of limitations inherent to the study design. Firstly, the study sample consisted of only healthy volunteers. The significance and validity of the VDM index should be further assessed in subjects with impaired fasting glucose or glucose intolerance and T2DM, so as to gain a better understanding of the role of vitamin D metabolism in states of normal and disturbed glucose homeostasis. In addition, all our subjects were of Asian descent, and all were non-obese (BMI <25 kg/m^2^). Accordingly, evaluating the relationship between vitamin D metabolism and metabolic function in overweight and obese subjects, and individuals of other ethnicities, will further advance our understanding of the role of vitamin D in obesity-related metabolic abnormalities, as well as in ethnic differences in metabolic function. Further, serum PTH was not monitored. PTH is known to facilitate the conversion of 25(OH)D_3_ to 1,25(OH)_2_D_3_, and it has been speculated that higher PTH levels reflect tissue-specific deficiency, given that low 25(OH)D_3_ is not always associated with higher serum PTH [16,30]. Finally, vitamin D supplementation was not part of the exclusion criteria, which could have resulted in some subjects having higher-than-normal serum 25(OH)D_3_. Taking into account the larger relative standard deviation value of 25(OH)D_3_ in high VDM subjects, we cannot preclude the possibility that a number of subjects in our sample may have had high intakes of vitamin D. We also considered our small sample size in this analysis. Wide variation in measurement accuracy among analytical methods has also been documented; therefore, caution should be taken when comparing serum values measured by mass spectrometry, as performed in our study, with values obtained from other techniques such as radioimmunoassays, which are the most common analytical methods for measuring 25(OH)D_3_.

## 5. Conclusions

Our findings raise important questions about the external validity of 25(OH)D_3_ measurements in determining vitamin D sufficiency for metabolic health outcomes. Our results suggest that altered vitamin D metabolism, likely linked to altered vitamin D signaling in tissues of physiological relevance to metabolic health (i.e., muscle, pancreas, and liver), is independently associated with derangements in glucose homeostasis. The associations between the VDM index and beta-cell function observed in the present study warrant a further investigation into the use of vitamin D metabolites as a biomarker of diabetes risk, as well as a rethinking of current strategies in the treatment of vitamin D deficiency.

## Figures and Tables

**Table 1:** Associations between the VDM index and cardiometabolic risk factors in all study subjects (*n* = 65).

*Subject Characteristics*	VDM Index			
	Unadjusted		Adjusted for Age, Sex, and BMI	
	*r*	*p*	ρ	*p*
Age	0.208	0.096		
BMI	−0.102	0.421		
Percent body fat (%)	−0.308 *	0.012	−0.261 *	0.041
Systolic blood pressure (mmHg)	0.054	0.671	−0.168	0.192
Diastolic blood pressure (mmHg)	−0.103	0.413	−0.251 *	0.049
Waist circumference (cm)	−0.075	0.551	−0.330 **	0.009
Triglyceride (mmol/L)	0.042	0.738	0.076	0.578
Cholesterol (mmol/L)	0.299 *	0.016	0.225	0.078
HDL-cholesterol (mmol/L)	0.139	0.269	0.200	0.119
LDL-cholesterol (mmol/L)	0.277 *	0.025	0.151	0.240
SAT (mL)	−0.278 *	0.025	−0.313 *	0.013
VAT (mL)	−0.043	0.734	−0.222	0.101
IHTG (%)	−0.161	0.201	−0.231	0.087
VO_2max_ (mL/kg∙min)	0.227	0.071	0.240	0.062
Insulin sensitivity	0.226	0.085	0.271 *	0.043
Total disposition index	0.311 *	0.012	0.281 *	0.028

BMI, body mass index; VDM, vitamin D metabolite; HDL, high-density lipoprotein; LDL, high-density lipoprotein; SAT, subcutaneous adipose tissue; VAT, visceral adipose tissue; IHTG, intrahepatic triglyceride; VO_2max_, maximum oxygen uptake. Insulin sensitivity was calculated as the glucose disposal rate adjusted for the steady-state plasma insulin concentration (M/I ratio), and the total disposition index was calculated as the product of the total insulin secretion rate area-under-the-curve and the M/I ratio. Data are shown as Pearson’s correlation coefficients (*r*) and partial coefficients after adjusting for age, sex, and BMI (ρ). Statistical significance was set at *p* < 0.05 (*) and *p* < 0.01 (**).

**Table 2:** Characteristics of subjects with a low and a high VDM index.

	High VDM Index (*n* = 17)	Low VDM Index (*n* = 17)	*p*
Age	41.3 ± 15.5	39.3 ± 13.6	0.692
BMI (kg/m^2^)	21.1 ± 1.3	22.0 ± 1.8	0.101
25(OH)D_3_ (ng/mL)	48.4 ± 13.2	45.6 ± 14.1	0.552
24,25(OH)_2_D_3_ (ng/mL)	23.3 ± 10.4	18.8 ± 8.6 *	0.015
1,25(OH)_2_D_3_ (ng/mL)	10.6 ± 4.2	6.1 ± 1.5 ***	<0.001
VDM index	2.32 ± 0.31	2.00 ± 0.27 **	0.003
Percent body fat (%)	27.5 ± 7.7	29.3 ± 7.5	0.508
Systolic blood pressure (mmHg)	124 ± 11	123 ± 11	0.976
Diastolic blood pressure (mmHg)	77 ± 10	82 ± 12	0.185
Waist circumference (cm)	70.4 ± 6.8	73.9 ± 7.2	0.159
Triglyceride (mmol/L)	0.80 ± 0.26	1.04 ± 0.52	0.098
Cholesterol (mmol/L)	5.47 ± 1.08	4.99 ± 0.91	0.175
HDL-cholesterol (mmol/L)	1.65 ± 0.42	1.48 ± 0.32	0.187
LDL-cholesterol (mmol/L)	3.46 ± 0.91	3.05 ± 0.78	0.169
HbA1c (mmol/mol)	35.3 ± 3.6	35.9 ± 3.1	0.576
Fasting plasma glucose (mg/dL)	87.8 ± 10.6	88.1 ± 8.1	0.923
Fasting plasma insulin (mU/L)	4.2 ± 2.4	5.9 ± 4.1	0.150
Fasting free fatty acids (mEq/L)	0.36 ± 0.15	0.38 ± 0.19	0.750
SAT (mL)	1691 ± 786	1947 ± 712	0.326
VAT (mL)	667 ± 424	940 ± 612	0.142
IHTG (%)	1.7 ± 1.3	4.2 ± 5.4	0.071
VO_2max_ (mL/kg∙min)	31.1 ± 9.8	35.4 ± 9.2	0.198

Subjects were matched for age, BMI, sex, and serum 25(OH)D_3_. BMI, body mass index; VDM, vitamin D metabolite; HDL, high-density lipoprotein; LDL, high-density lipoprotein; SAT, subcutaneous adipose tissue; VAT, visceral adipose tissue; IHTG, intrahepatic triglyceride; VO_2max_, maximum oxygen uptake; 25(OH)D_3_, 25-hydroxyvitamin D_3_; 1,25(OH)_2_D_3_, 1α,25-dihydroxyvitamin D_3_; 24,25(OH)_2_D_3_, *24R*,25-dihydroxyvitamin D_3_. Data are the mean ± SD. Statistical significance was set at *p* < 0.05 (*), *p* < 0.01 (**) and *p* < 0.001 (***), compared with subjects in the high VDM index group.
